# Purinergic regulation of angiogenesis by human breast carcinoma-secreted nucleoside diphosphate kinase

**DOI:** 10.1038/sj.bjc.6604019

**Published:** 2007-10-16

**Authors:** S M Rumjahn, M A Javed, N Wong, W E Law, I L O Buxton

**Affiliations:** 1 Department of Pharmacology MS318, University of Nevada School of Medicine, 1664 N Virginia Street, Reno, NV 89557, USA

**Keywords:** breast cancer, tumour angiogenesis, nucleoside diphosphate kinase, polyphenolic compounds, P2Y receptors

## Abstract

MDA-MB-435S human breast cancer cells (435S) secrete nucleoside diphosphate kinase (NDPK) that supports metastases and is inhibited by epigallocatechin gallate (EGCG) and ellagic acid (EA). We hypothesise that 435S cell-secreted NDPK-B supports tumour formation by modulating ATP levels locally to activate endothelial cell (EC) P2Y receptor-mediated angiogenesis. Epigallocatechin gallate (IC_50_=8–10 *μ*M) and EA (IC_50_=2–3 *μ*M) suppressed 435S cell growth, but had less effect on human CD31^+^ EC growth. Epigallocatechin gallate (IC_50_=11 *μ*M) and EA (IC_50_=1 *μ*M) also prevented CD31^+^ EC tubulogenesis on Matrigel™. 435S cell-conditioned media induced tubulogenesis in a cell number, time, and nucleotide-dependent manner. Ellagic acid (1 *μ*M), but not equimolar EGCG, reduced cell number-dependent angiogenesis. P2Y_1_ receptor activation by NDPK-generated nucleotide (100 *μ*M ATP) or by 10 *μ*M 2-methyl-thio-ATP (2MS-ATP) promoted tubulogenesis on collagen and was blocked by the P2Y_1_ antagonist MRS2179 (10 *μ*M). Physiological amounts of purified as well as 435S cell-secreted NDPK also promoted angiogenesis that was attenuated by NDPK depletion or 10 *μ*M MRS2179, indicating a P2Y_1_ receptor-mediated pathway. These results support the notion that secreted NDPK mediates angiogenesis via P2Y receptor signalling and suggests that novel inhibitors of NDPK may be useful as therapeutics.

The onset of tumour vascularisation, the ‘angiogenic switch’, supplies the tumour with nutrients and oxygen that promote growth. It is known that tumours in a pre-vascularised state are unable to grow beyond 2–3 mm^3^ (approximately 10^5^–10^6^ cells) in size where a balance between active proliferation and apoptosis keeps the tumour in an arrested state of growth (Holmgren *et al*, 1995). Conventional treatment modalities targeting the genetically instable cancer cells are burdened with toxicity and the emergence of drug-resistant cell types. Directly targeting the genetically stable vascular endothelial cells (ECs) involved in tumour-mediated angiogenesis has shown promise when co-administered with conventional cancer therapies (Clinical Trials, 2006). It has been suggested that angiogenic therapies initially normalise the vasculature and thus sensitise tumours to concurrently administered chemo/radiotherapies resulting in synergistic outcomes (Jain, 2001; Tong *et al*, 2004). Since women who succumb to breast cancer have often undergone surgery to remove the primary tumour, antiangiogenic therapies may be considered essential in combating the metastatic spread often seen years later.

Nucleoside diphosphate kinase (NDPK) (EC 2.7.4.6), the protein product of gene *nm23*, was first described as an intracellular ‘housekeeping’ enzyme, which covalently transfers the *γ*-phosphate from a nucleoside triphosphate to a nucleoside diphosphate. While expression of *nm23-H1* (NDPK-A) and *nm23-H2* (NDPK-B) have been examined, less attention to their enzymatic function in cancer and metastasis has been considered. Nucleoside diphosphate kinase is known to be distributed in the cytosol and plasma membrane of cells, as well as the nucleus (Bertheau *et al*, 1994) where the NDPK-B isoform functions as PuF, a c-*MYC* transcription factor (Postel, 2003). Non metastatic clone 23 gene, *nm23*, was first observed in murine carcinoma cell lines and said to negatively correlate with a cancer's metastatic potential (Roymans *et al*, 2002), although there is evidence to the contrary (Postel, 2003). We have shown that both MDA-MB-435S and -231 metastatic human breast carcinoma cells secrete NDPK-B into their surrounding environment when cultured *in vitro* (Anzinger *et al*, 2001). Nucleoside diphosphate kinase is secreted, despite the lack of a signal sequence, probably via non-classical export as has previously been reported with proangiogenic fibroblast growth factors 1 and 2 (FGF-1 and FGF-2) (Nickel, 2003). The transphosphorylation activity of NDPK-B has been reported to promote the metastatic potential of human melanoma cells (Hamby *et al*, 2000).

The anticarcinogenic potential of catechins (polyphenols found in green tea) was first observed and reported over a decade ago. Epigallocatechin gallate (EGCG) has been identified as the major catechin in green tea and shown to inhibit both the growth of various cancers (Liang *et al*, 1999; Suganuma *et al*, 1999), and angiogenesis (Cao and Cao, 1999; Singh *et al*, 2002). We have previously shown that EGCG and ellagic acid (EA) are more potent inhibitors of NDPK activity than known nucleoside analogues (Malmquist *et al*, 2001) and have suggested a role for NDPK inhibition in the management of metastasis (Anzinger *et al*, 2001). A role for NDPK activity in blood vessel regulation is known. The Nucleotide Axis Hypothesis posits that ecto-NDPK on vascular ECs facilitates the regulation of extracellular nucleotide levels (e.g., ATP) (Buxton *et al*, 2001; Kaiser and Buxton, 2002). Since perturbing the regulation of nucleotide levels has been shown to impair angiogenesis (Goepfert *et al*, 2001; Moser *et al*, 2001), we hypothesise that extracellular NDPK takes an advantage of nucleotide signalling on vascular ECs and promotes pathological angiogenesis.

Heterotrimeric G-protein-coupled ATP receptors (P2Y) are recognised as integral modulators of platelet aggregation and blood flow regulation. Extracellular ATP activates P2Y receptors on vascular ECs to release vasoactive mediators such as nitric oxide, prostacyclin, and additional ATP (Yang *et al*, 1994; Buxton *et al*, 2001) which can also elicit angiogenic effects (Kashiwagi *et al*, 2005). Here, we provide evidence that human breast cancer cell-secreted NDPK promotes EC tubulogenesis *in vitro*. This supports our hypothesis that NDPK-mediated increases in extracellular ATP levels by breast tumour cells provide a mechanistic basis by which targeted therapeutics can prevent tumour growth and angiogenesis.

## MATERIALS AND METHODS

### Cell culture

The human breast cancer cell line MDA-MB-435S (435S) was purchased from the American Type Culture Collection (Manassas, VA, USA). Human cardiac ECs were previously isolated by fluorescence-activated cell sorting (FACS) for CD31 (PECAM) and immortalised by human telomerase reverse transcriptase (hTRT) – referred to as CD31^+^ cells. Unless specifically stated, both cell types were maintained and incubated in Dulbecco's modified Eagle's medium (DMEM) (HyClone, Logan, UT, USA) supplemented with 10% fetal bovine serum (FBS) (Atlanta Biological, Lawrenceville, GA, USA), penicillin–streptomycin, (1500 U l^−1^–100 mg l^−1^) and 0.5 mg l^−1^ Fungizone (Invitrogen, Carlsbad, CA, USA) at 37°C in a humidified 5% CO_2_/95% air atmosphere.

### Effect of EGCG and EA on MDA-MB-435S and CD31^+^ cell growth

To determine if EGCG and/or EA (Sigma, St Louis, MO, USA) inhibit 435S cell growth, 1.2 × 10^5^ cells per well were seeded onto six-well tissue culture plates and incubated in the presence of EGCG or EA (0.3–30 *μ*M) for 2–5 days. For longer incubation periods (i.e., days 4 and 5) culture media were replaced after 72 h with fresh media containing appropriate ECGC or EA concentrations. To determine if EGCG and/or EA inhibit CD31^+^ cell growth, 9.6 × 10^4^ cells per well were first seeded onto six-well tissue culture plates and incubated for 24 h. Culture media were then replaced with fresh media containing EGCG or EA (0.3–10 *μ*M) and incubated for 24 h. At desired treatment end points, EGCG and EA (2–5 days and 24 h, respectively) treated cells were counted using a Coulter Z1 cell counter (Coulter Corp, Hialeah, FL, USA) and compared to non-treatment controls.

### *In vitro* angiogenesis scoring technique

**Figure 1:**
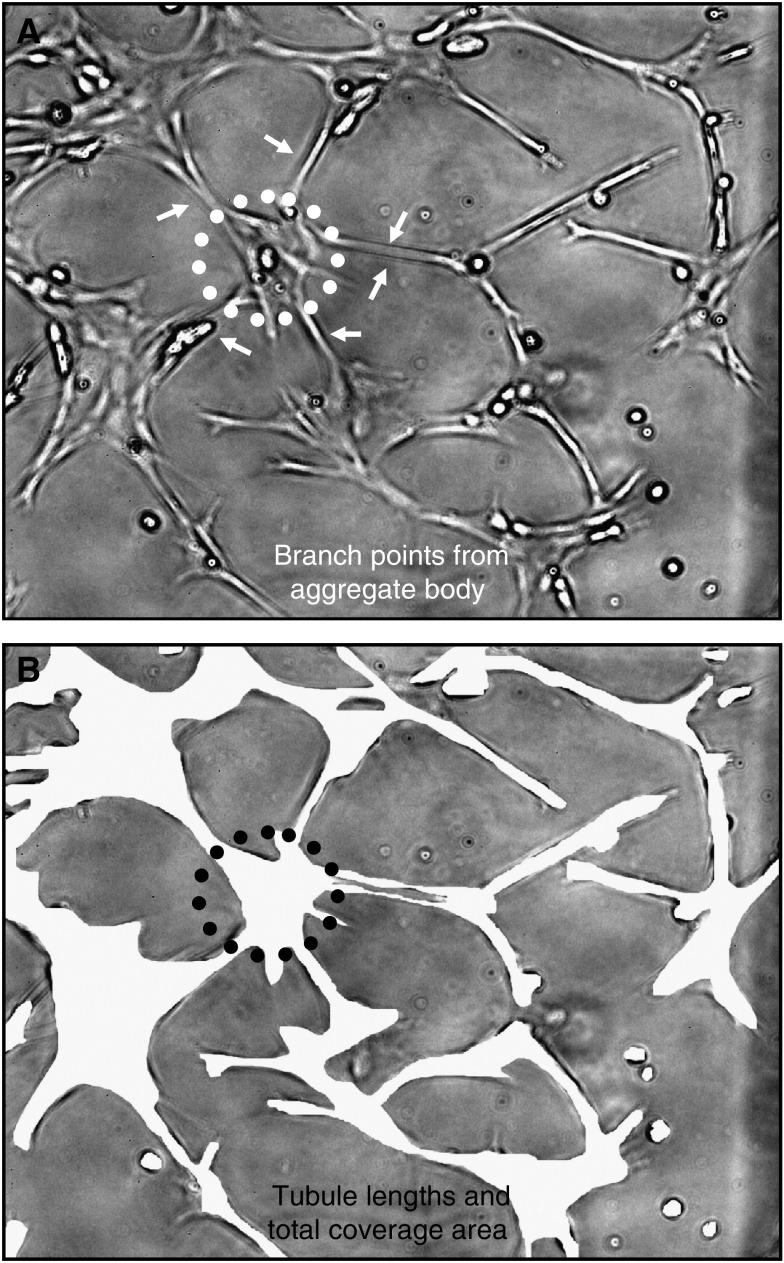
Angiogenesis scoring. (**A**) Tubule lengths (*l*) and number of branch points (*bp*). (**B**) Endothelial cell (EC) surface area (*a*). Relative angiogenesis score (*s*)=*bp* × *l × a*. Photographs were imaged at a magnification of × 100.

CD31^+^ cell tubulogenesis (i.e., tubule-like formation in culture) was quantified using a high-resolution digital camera (FujiPro 2; 12 mega pixel) attached to a Nikon 300 inverted microscope to document CD31^+^ cell tubule-like formations. An angiogenesis score was obtained by analysing digital images (× 100) collected from the central pointing corners of quadrants I–IV in each well and averaging the four scores. Each angiogenesis score represents the product of mean number of branch points multiplied by mean branch length multiplied by mean cell surface area (illustrated in Figure 1). Morphometry (in pixels) was performed using MetaMorph image analysis software (V4.01; Universal Imaging Corporation, Downingtown, PA, USA). The lengths of tubule-like formations were measured for all completely visible connections between edges of aggregate bodies (Figure 1A). The number of branch points connecting one aggregate body to other aggregate bodies (usually 1–3) was counted when the complete connection was within view of the image (Figure 1A). The total CD31^+^ cell surface area was also determined from these images (Figure 1B). Number of branch points (*bp*), length (*l*), and cell surface area (*a*) were multiplied to produce a relative angiogenesis score (*s*). Thus, *s*=*bp* × *l* × *a*.

### Effect of EGCG and EA on angiogenesis

Four-well glass slide chambers were coated with a thin layer (∼10 *μ*l) of Matrigel™ (BD Biosciences, Bedford, MA, USA) and allowed to solidify at 37°C/5% CO_2_ for 30 min. To determine if EGCG and/or EA inhibit CD31^+^ cell tubulogenesis, 5 × 10^4^ cells were first seeded onto four-well glass slide chambers coated with Matrigel and allowed to attach for 30 min at 37°C/5% CO_2_. The test substances EGCG or EA (0.3–30 *μ*M) were then added to the individual wells as appropriate and incubated with CD31^+^ cells for 5.5 h. The antiangiogenic drug endostatin (Sigma), 0.1–1.0 *μ*g ml^−1^, was used as a positive control. Non-treatment controls were performed for normalisation and comparison of EGCG and EA effects on *in vitro* angiogenesis.

### Effect of MDA-MB-435S cell-conditioned media on angiogenesis

To determine if secreted molecules from 435S cells promote CD31^+^ *in vitro* angiogenesis, 435S cells were seeded onto Transwell™ tissue culture inserts (3.0 *μ*m pores; Corning, Acton, MA, USA) for incubation times up to 24 h. These various conditioned media were examined via the above described CD31^+^ cell Matrigel tubulogenesis assay using 24-well tissue culture plates. Briefly, 435S cell number (1.5–6 × 10^4^ cells incubated for 12 h) and conditioning time (6 × 10^4^ cells incubated for 30 min to 24 h) were used as experimental variables. At their incubation end points, the various conditioned media were frozen at −30°C and later assessed for their effects on *in vitro* angiogenesis.

### Effect of EGCG and EA on MDA-MB-435S cell-conditioned media promoted angiogenesis

The conditioned media from 1.5 × 10^4^ to 6 × 10^4^ 435S cells incubated for 12 h was further investigated with the addition of either 1 *μ*M EGCG or EA. These experiments were conducted using the CD31^+^ cell Matrigel tubulogenesis assay on 24-well tissue culture plates. Endostatin (1.0 *μ*g ml^−1^) was used as a positive antiangiogenic control. Non-treatment controls were performed for normalisation and comparison.

### Effect of apyrase on MDA-MB-435S cell-conditioned media promoted angiogenesis

The conditioned media from 6 × 10^4^ 435S cells incubated for 60 min was also further investigated with the addition of potato apyrase (grade 1; Sigma; EC 3.6. 1.5). Briefly, the 435S cell-conditioned media were incubated in a final concentration of 0.75 U apyrase per millilitre conditioned media for 5 min at 37°C (1 U liberates 1.0 *μ*mol orthophosphate from ATP or ADP per min at pH 6.5, 30°C). This conditioned media pretreated with apyrase was then observed in the CD31^+^ cell Matrigel tubulogenesis assay onto 24-well tissue culture plates. Non-treatment controls were performed for normalisation and comparison.

### Effect of ATP and 2-methyl-thio-ATP on angiogenesis

The following experiments were performed with a lower concentration of FBS supplementation (2 *vs* 10%) and collagen instead of Matrigel to rule out the role of angiogenic factors present in Matrigel to more clearly distinguish the amount of proangiogenic stimulation attributed to P2Y receptor activation alone. To determine if 2-methyl-thio-ATP (2MS-ATP) and/or ATP (Sigma) stimulate *in vitro* angiogenesis, 3 × 10^4^ CD31^+^ cells per well were first seeded onto 24-well tissue culture plates coated with 1 mg ml^−1^ collagen (Rat type I; BD Biosciences) and allowed to attach for 30 min. The P2Y receptor agonists 2MS-ATP (P2Y_1_R; 10 *μ*M) and ATP (P2Y_1/2_R; 100 *μ*M) were added to their respective wells and incubated with CD31^+^ cells for 24 h. CD31^+^ cell tubulogenesis was also observed in the presence of 10 *μ*M MRS2179 (P2Y_1_R antagonist; Sigma) with either 10 *μ*M 2MS-ATP or 100 *μ*M ATP. Endothelial growth medium-2 (EGM-2™; Clonetics, East Rutherford, NJ, USA) was used as a positive control to confirm that this modified assay could successfully detect angiogenic stimulation. Non-treatment controls were performed for normalisation and comparison. This experiment was repeated with 2 × 10^4^ CD31^+^ cells per well and incubated for a longer duration of 72 h.

### Preparation of human breast carcinoma secreted NDPK extract

MDA-MB-435S cells were grown to ∼75% confluence in T150 tissue culture flasks and then washed × 3 with room air Krebs buffer (RAK) containing (in mM) 120 NaCl, 5 KCl, 0.587, KH_2_PO_4_, 0.589 Na_2_HPO_4_, 2.5 MgCl_2_, 20 *α*-D-glucose, 2.5 CaCl_2_, 25 Tris, and 5 NaHCO_3_. The cells were then slowly rocked and incubated in RAK at 37°C for 90 min. This RAK containing secreted NDPK-B was concentrated for 30 min at 4°C and 2000 **g** using Amicon Ultra-15 10 kDa Centrifugal Filters (Millipore Corporation, Bedford, MA, USA). The remaining liquid captured above the 10-kDa mark was once again filtered as described above. The concentrated (∼200-fold) 435S cell-secreted NDPK extract was frozen at (−80°C) and used in subsequent angiogenesis experiments. The average protein concentration for 1 × NDPK extract (19.23 *μ*g ml^−1^) was used to mimic the approximate amount of secreted NDPK-B seen in the conditioned media of ∼75% confluent 435S carcinoma cell cultures. Nucleoside diphosphate kinase transphosphorylation activity; ATP production from GTP donor and ADP acceptor was measured using a luciferin–luciferase ATP detection assay (Sigma). Using only ADP and no GTP donor in our activity assay showed no ATP production, indicating that adenylate kinase activity was not responsible for the ATP production observed (not shown).

### Effect of purified NDPK on angiogenesis

To determine if purified NDPK promotes *in vitro* angiogenesis, 3 × 10^4^ CD31^+^ cells per well were first seeded onto 24-well tissue culture plates coated with 1 mg ml^−1^ collagen (Rat type I) and allowed to attach for 30 min. Semi-purified bovine liver NDPK (Sigma) and subsequent affinity purified NDPK transphosphorylation activity levels; as measured using a luciferin–luciferase ATP detection assay were used to match the activity level of 1 × 435S cell-secreted NDPK extract. This purified NDPK with or without nucleotide donor and acceptor (300 *μ*M GTP and 30 *μ*M ADP, respectively) were then added to wells and incubated with the CD31^+^ cells for 24 h. EGM-2 was used as a positive control, while non-treatment controls were performed for normalisation and comparison.

### Effect of MRS2179 on NDPK extract induced angiogenesis

To determine if MRS2179 (P2Y_1_R antagonist) reduces the degree which 435S cell-secreted NDPK extract promotes *in vitro* angiogenesis, 3 × 10^4^ CD31^+^ cells per well were first seeded onto 24-well tissue culture plates coated with 1 mg ml^−1^ collagen (Rat type I) and allowed to attach for 30 min. 1 × NDPK extract with or without 10 *μ*M MRS2179 was then incubated with the CD31^+^ cells for 24 h. EGM-2 was used as a positive control, while non-treatment controls were performed for normalisation and comparison. This experiment was also repeated with 2 × 10^4^ CD31^+^ cells per well in experiments lasting 72 h.

### Statistical analyses

All graphs were prepared using Prism Graphing Software (V4.03; GraphPad Software, San Diego, CA, USA) and statistical analyses were performed using InStat Statistical Software (V3.0; GraphPad Software), with *P*<0.05 considered to be statistically significant. All growth and angiogenesis scores were tested for statistical significance using ANOVA and Kruskal–Wallis multiple comparisons post-test unless otherwise stated. Data points and error bars represent means±s.e.m. *P<0.05; **P<0.01; ***P<0.001 (*vs* negative control); ^+^*P*<0.05 (EGCG *vs* EA); ^#^*P*<0.05; ^##^*P*<0.01 (*vs* 435S cell stimulation).

## RESULTS

### Epigallocatechin gallate and EA suppress MDA-MB-435S cell growth

**Figure 2:**
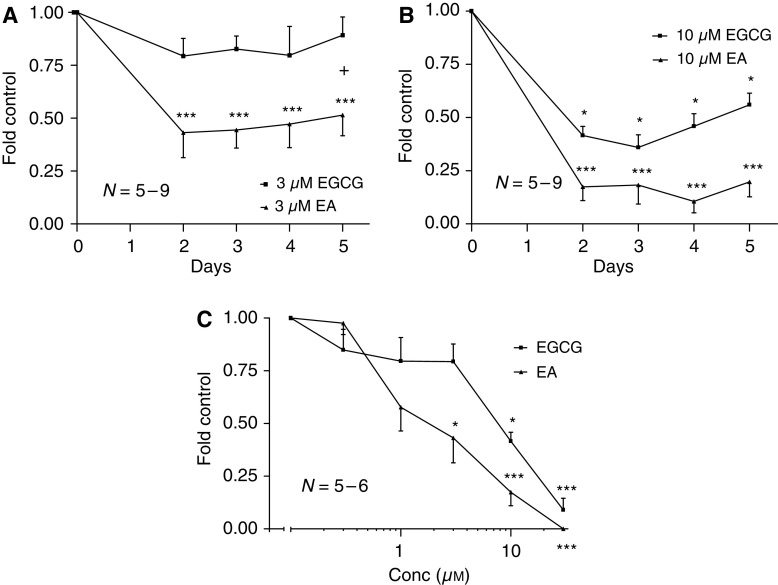
Epigallocatechin gallate (EGCG) and ellagic acid (EA) inhibit human breast cancer growth *in vitro*. (**A** and **B**) EA treatment reduced cell number when compared to equimolar amounts of EGCG. (**C**) EA treatment over 2 days reduced cell number at one half-log higher potency than EGCG. Control; 435S cells incubated in CDMEM supplemented with 10% FBS. Control mean=212 806±35 642 435S cells.

435S cells incubated with 3 *μ*M EGCG over 5 days inhibited growth ∼10–20%, while equimolar EA was more effective with ∼50–60% growth suppression. Ellagic acid but not EGCG significantly inhibited cell growth at 3 *μ*M when compared to non-treatment controls (*P*⩽0.001; Figure 2A). The addition of 10 *μ*M EGCG for 5 days inhibited growth ∼45–65%, while equimolar EA was more effective producing ∼80–90% suppression. Both EA and EGCG treatments significantly impeded cell growth at 10 *μ*M when compared to non-treatment controls (*P*⩽0.05; Figure 2B). 435S cells incubated with 0.3–30 *μ*M EGCG or EA over 2 days produced dose-dependent inhibition of growth with an apparent IC_50_ for EGCG of ∼8 *μ*M and an IC_50_ for EA of ∼2 *μ*M (day 5 EGCG IC_50_ of ∼10 *μ*M and EA IC_50_ of ∼3 *μ*M; data not shown). Treatment with EA⩾1 *μ*M over 2 days significantly suppressed cell growth when compared to non-treatment control (*P*⩽0.05; Figure 2C) and at a ∼1/2 log higher potency than EGCG (3 *μ*M *vs* 10 *μ*M=approximately 60% inhibition). At micromolar concentrations, both agents were cytostatic rather than cytotoxic since cells regrew when drug was removed.

### Epigallocatechin gallate and EA suppress human EC growth and tubule-like formations on Matrigel

**Figure 3:**
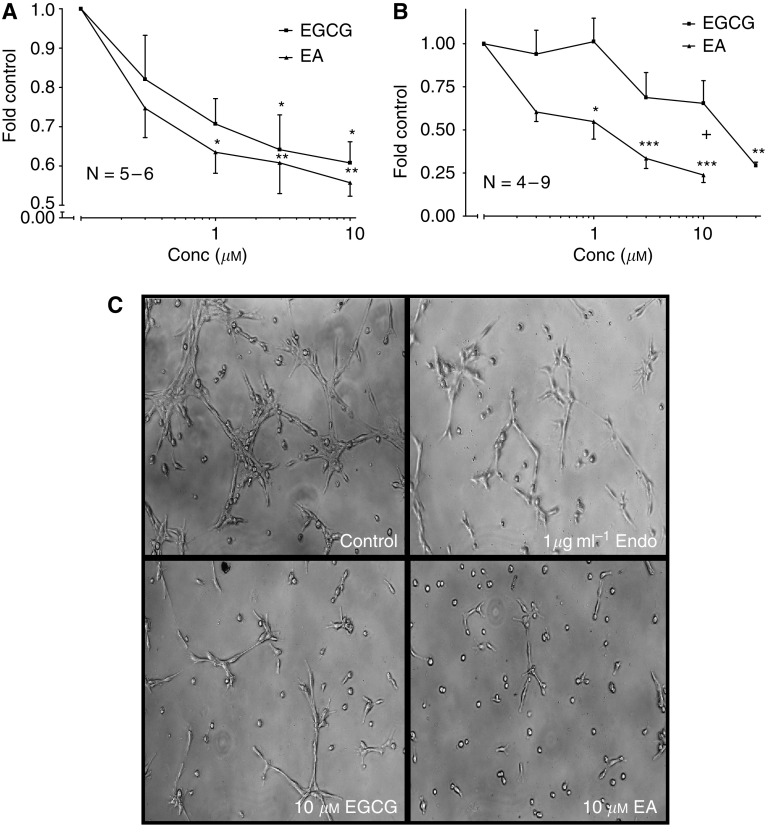
Epigallocatechin gallate (EGCG) and ellagic acid (EA) inhibit human EC growth and angiogenesis *in vitro*. (**A**) Both EGCG and EA reduced CD31^+^ cell number when incubated for 24 h at ⩾1 *μ*M. Control *A* mean=163 094±27 859 CD31^+^ cells. (**B**) EA was more potent than EGCG in reducing CD31^+^ cell tubulogenesis over 6 h. Control *B* mean=12 078.1±3355.3 angiogenesis units. (**C**) Representative images illustrating endostatin, EGCG, and EA inhibition of tubule-like formations (× 100 magnification). Control; CD31^+^ cells incubated in CDMEM supplemented with 10% FBS.

CD31^+^ cells incubated with 0.3–10 *μ*M EGCG or EA for 24 h produced dose-dependent inhibition of growth. The addition of EGCG or EA significantly suppressed growth ∼35–45% at concentrations ⩾3 *μ*M when compared to non-treatment control (*P*⩽0.05; Figure 3A). CD31^+^ cells incubated on Matrigel with 0.3–30 *μ*M EGCG or EA produced dose dependent inhibition of *in vitro* angiogenesis with an apparent IC_50_ for EGCG of ∼11 *μ*M and a more potent IC_50_ for EA of ∼1 *μ*M. Addition of EA significantly reduced angiogenesis when compared to non-treatment control (*P*⩽0.01; Figure 3B and C). Unlike growth inhibition, EA suppressed CD31^+^ cell tubulogenesis at approximately one log lower concentration than EGCG (3 *μ*M *vs* 30 *μ*M=∼70% inhibition). Endostatin, the antiangiogenic control, produced dose dependent and significant inhibition of angiogenesis at 1 *μ*g ml^−1^ comparable to concentrations ⩾3 *μ*M of EGCG and EA (*P*⩽0.05; data not shown).

### MDA-MB-435S conditioned media promotes *in vitro* angiogenesis in a time-, cell number-, and nucleotide-dependent manner

**Figure 4:**
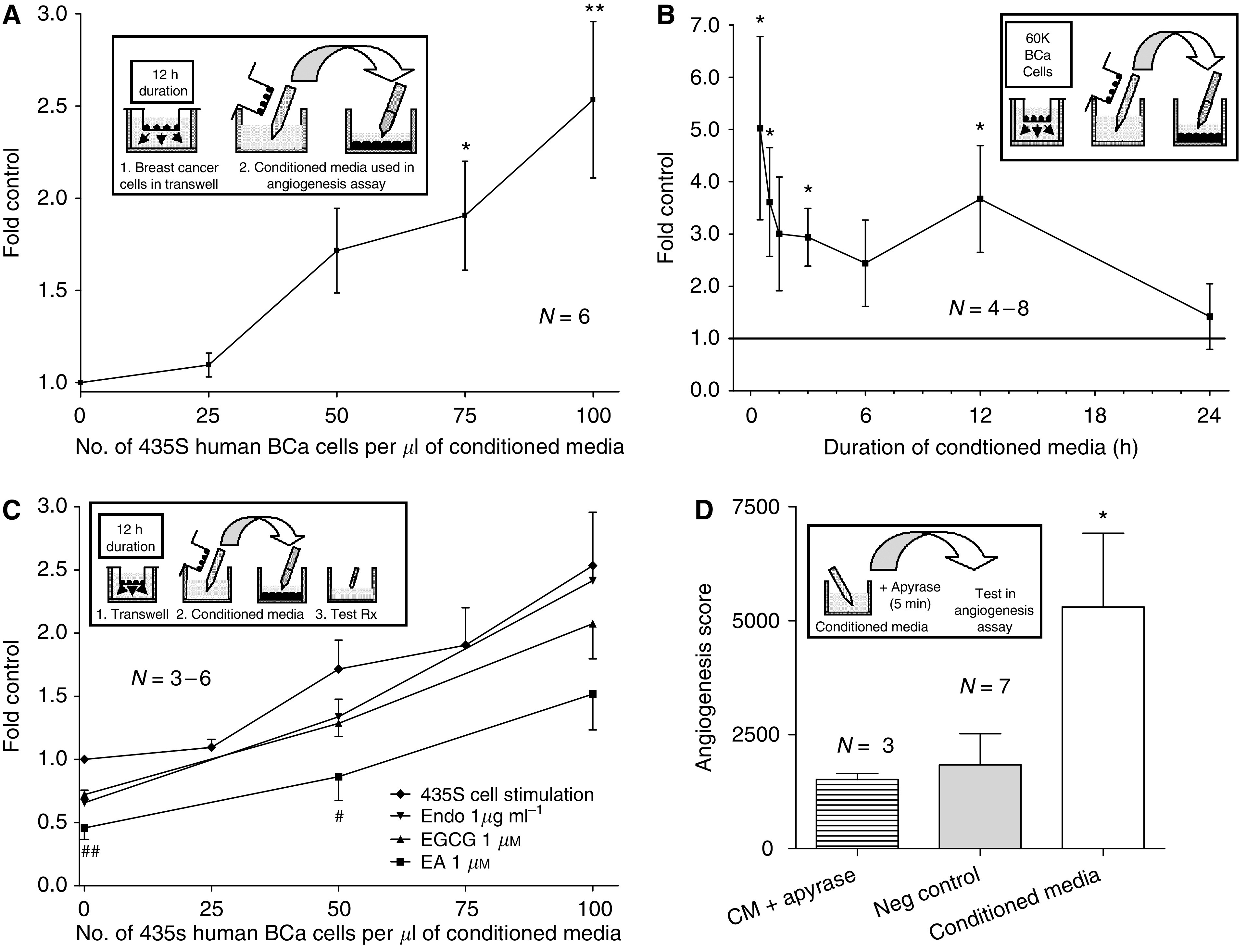
Human breast cancer cell-conditioned media promotes *in vitro* angiogenesis. CD31^+^ EC were incubated on Matrigel™ with media conditioned by MDA-MB-435S cells (varying conditioning time or cell number) and tubulogenesis was observed after 6 h. (**A**) increasing 435S cell number induced *in vitro* angiogenesis. Control *A* consisted of CDMEM supplemented with 10% FBS and conditioned for 12 h with no 435S cells. Control mean=2581.5±374.6 angiogenesis units. (**B**) shorter 435S cell conditioning time stimulated *in vitro* angiogenesis. Control *B* consisted of CDMEM conditioned for 24 h with no 435S cells. Control mean=3373.0±1179.3 angiogenesis units. (**C**) Ellagic acid (EA) but not epigallocatechin gallate (EGCG) or endostatin treatment diminished 435S cell number dependent *in vitro* angiogenesis. Control *C* consisted of CDMEM conditioned for 24 h with no 435S cells. Control mean=12 229.1±5573.4 angiogenesis units. (**D**) Addition of apyrase to 435S cell-conditioned media blocked its ability to stimulate *in vitro* angiogenesis. Control *D* consisted of CDMEM conditioned for 60 min with no 435S cells.

We examined the effect of 435S cell-conditioned media on CD31^+^ cell tubulogenesis and its inhibition by polyphenolic compounds. Media containing secreted NDPK-B (conditioned media) were collected using a Transwell™ apparatus seeded with 435S cells. CD31^+^ cells incubated with 12-h conditioned media (varying 435S cell number from 1.5 × 10^4^ to 6 × 10^4^) on Matrigel demonstrated a progressive induction of angiogenesis with increasing numbers of 435S cells. A ratio of ⩾75 MDA-MB-435S cells per microlitre (4.5 × 10^4^ cells) significantly promoted angiogenesis at least two-fold when compared to control media conditioned without breast cancer cells (*P*⩽0.05; Figure 4A). CD31^+^ cells incubated with media conditioned by 6 × 10^4^ 435S cells (varying conditioning times – 30 min to 24 h) on Matrigel indicated an initial ∼5-fold induction burst of *in vitro* angiogenesis, which eventually returned to control-like levels after 24 h. Generally, conditioning times up to 12 h significantly promoted CD31^+^ cell tubulogenesis (*P*⩽0.05; Figure 4B).

The promotion of tubulogenesis by 12 h 435S cell-conditioned media (varying cell number) was diminished ∼45–60% by 1 *μ*M EA, ∼20–30% by 1 *μ*M EGCG, and ∼5–35% by 1 *μ*g ml^−1^ endostatin. Only the addition of EA to 435S-conditioned medium significantly reduced this cell number-dependent angiogenesis relative to controls (*P*⩽0.05; Figure 4C). Using a luciferin–luciferase ATP detection assay we also determined that 0.1–1.0 *μ*g ml^−1^ endostatin, our antiangiogenic control, did not inhibit the activity of 435S-secreted NDPK-B (data not shown) in contrast to EGCG and EA. Overall, 1 *μ*M EA produced better inhibition of *in vitro* angiogenesis induced by MDA-MB-435S cells than equimolar EGCG.

The observed promotion of tubulogenesis by 60-min conditioned media (6 × 10^4^ 435S cells; *P*⩽0.05; Figure 4D) was attenuated back to control levels with the addition of 0.75 U apyrase per ml media. This degradation of nucleotides (i.e., ATP and ADP) by the apyrase enzyme blocked the ∼2.9-fold induction above controls. The addition of apyrase to non-conditioned control media had no effect (data not shown).

### Purified and MDA-MB-435S secreted NDPK induce angiogenesis

**Figure 5:**
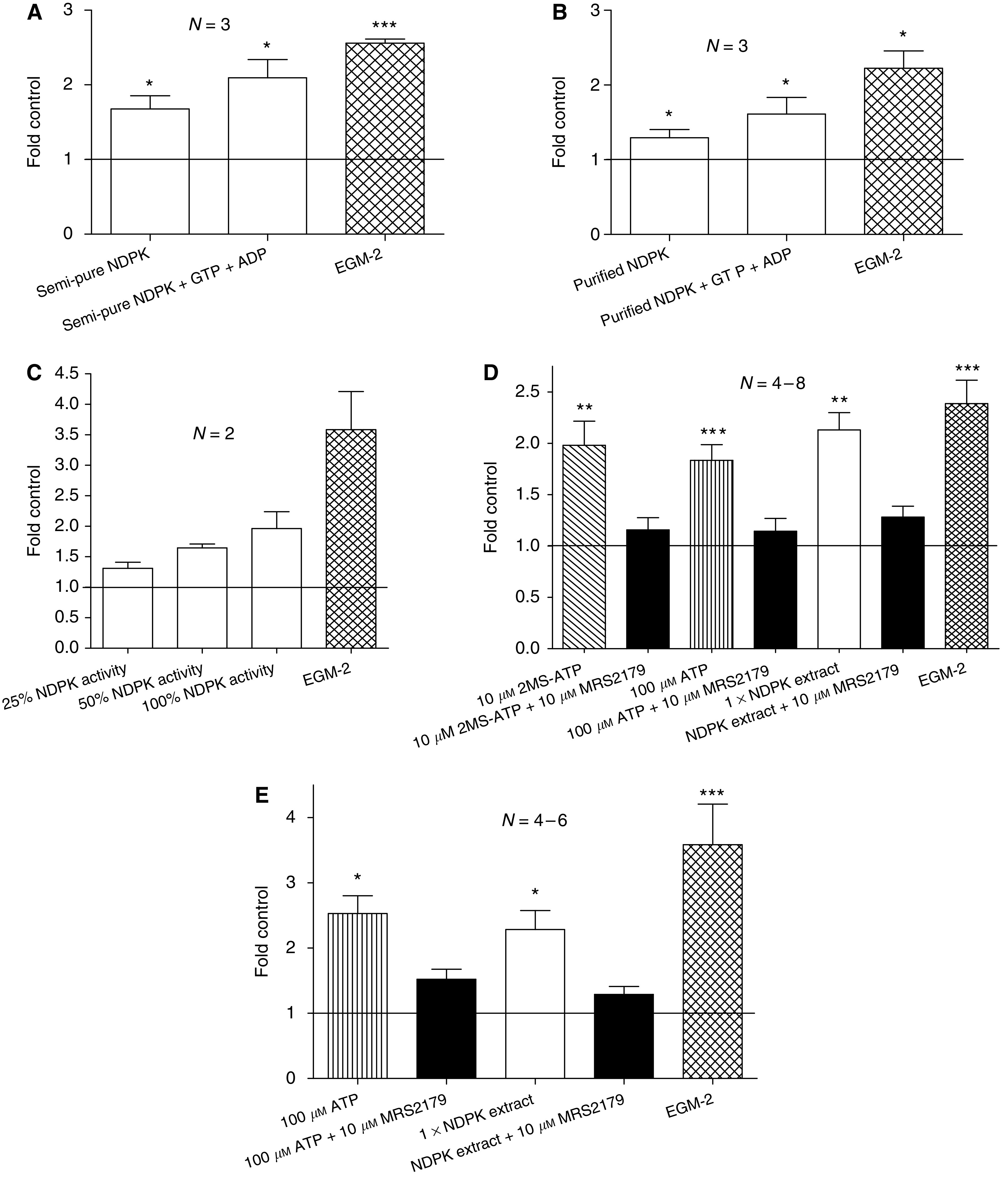
Extracellular nucleoside diphosphate kinase (NDPK) and EC P2Y_1_ receptor activation induce angiogenesis *in vitro*. (**A** and **B**) semi-pure as well as purified bovine liver NDPK induced CD31^+^ cell tubulogenesis over 24 h. Statistical significance versus control was determined using the Mann-Whitney test. (**C**) NDPK-B derived from 435S cell-secreted NDPK extract promoted *in vitro* angiogenesis over 72 h at various depletion levels. Control *C* mean=2645.9 angiogenesis units. (**D**) 24 h incubation with ATP, 2MS-ATP, and NDPK extract all stimulated angiogenesis; blocked by MRS2179. Control *D* mean=6183.8±1840.4 angiogenesis units. (**E**) 72 h incubation with ATP and NDPK extract both promoted angiogenesis; blocked by P2Y_1_R antagonist, MRS2179. Control *E* mean=1256.6±240.9 angiogenesis units. Controls consisted of CD31^+^ cells incubated in CDMEM supplemented with 2% FBS. The angiogenic stimulation control used was endothelial growth media-2 (EGM-2™) containing VEGF.

Incubation of CD31^+^ cells for 24 h with semi-pure and purified bovine liver NDPK (activity matched to 1 × 435S cell-secreted NDPK extract) increased *in vitro* angiogenesis at least ∼1.7- and ∼1.3-fold above control levels, respectively (Figure 5A and B). Angiogenic stimulation control EGM-2 further stimulated tubulogenesis two-fold above control. The semi-pure bovine liver NDPK was purified ∼12.5-fold, based on specific transphosphorylation activities, using an EDA-ATP sepharose column (Jena Bioscience, Germany). The combination of bovine liver NDPK, GTP (donor), and ADP (acceptor) induced tubulogenesis greater than that of NDPK alone. GTP and ADP alone induced a modest increase in tubulogenesis consistent with P2Y receptor activation (data not shown).

Progressive immunodepletion of NDPK-B from the 435S cell-secreted NDPK extract suppressed CD31^+^ cell tubulogenesis on collagen up to 65% when compared to non-depleted extract (Figure 5C), the depleted transphosphorylation activities were measured and compared to starting material using the luciferin–luciferase ATP detection assay. The significant increase in angiogenesis induced by 435S cell-secreted NDPK extract at 24 h was similar to the ∼2.0-fold stimulation seen with 10 *μ*M 2MS-ATP or 100 *μ*M ATP (*P*⩽0.01; Figure 5D) and blocked by 10 *μ*M MRS2179 (∼1.2-fold control). The 72-h assay produced a ∼2.3-fold increase in tubulogenesis after the addition of NDPK extract (*P*⩽0.05; Figure 5E) and a reduction back to ∼1.3-fold in the presence of 10 *μ*M MRS2179.

### P2Y receptor activation on human ECs stimulates angiogenesis

CD31^+^ cells incubated for 24 h with 10 *μ*M 2MS-ATP (P2Y_1_R agonist) or 100 *μ*M ATP (P2Y_1/2_R agonist) indicate a similar, significant ∼2.0-fold induction of *in vitro* angiogenesis above control levels comparable to the angiogenic stimulation control EGM-2 (containing VEGF) (*P*⩽0.001; Figure 5D). CD31^+^ cells incubated for 72 h with 100 *μ*M ATP demonstrate a significant ∼2.5-fold induction of tubulogenesis above control levels, while the EGM-2 control produced a ∼3.6-fold increase over control (*P*⩽0.05; Figure 5E). The use of 100 *μ*M 2MS-ATP and 1 mM ATP produced no increase in stimulation over 10 *μ*M 2MS-ATP or 100 *μ*M ATP alone (data not shown). The addition of 10 *μ*M MRS2179 (P2Y_1_R antagonist) to either 10 *μ*M 2MS-ATP or 100 *μ*M ATP stimulations diminished tubulogenesis back to near control levels (Figure 5D) after 24 h, while the addition of this P2Y_1_R antagonist over a 72 h period only suppressed ATP angiogenic stimulation back down to ∼1.5-fold that of control (Figure 5E). MRS2179 (10 *μ*M) showed no effect on tubulogenesis by itself (data not shown). We chose 10 *μ*M MRS2179 as maximal inhibition of P2Y_1_ receptor activation on ECs, consistent with our findings and those of others (Boyer *et al*, 1998; Baurand *et al*, 2001; Kaiser and Buxton, 2001, 2002) in EC systems. Varying concentrations of MRS2179 (0.1–10 *μ*M) did not affect NDPK transphosphorylation activity (data not shown), indicating P2Y_1_R antagonism as the primary mode of angiogenic inhibition.

## DISCUSSION

Our results demonstrate that both EA and EGCG inhibit MDA-MB-435S human breast cancer cell growth in a dose-dependent manner, consistent with the results of others (Suganuma *et al*, 1999; Seeram *et al*, 2005). Both EGCG and EA are near equipotent in suppressing CD31^+^ EC *in vitro* angiogenesis and 435S cell growth, while less potent in inhibiting CD31^+^ EC growth. The disparate effect of polyphenols between EC growth inhibition and tubulogenesis suppression suggests that P2Y signalling may be critical in the complex process of tubulogenesis, while EC growth could be less dependent on P2Y mediated effects of these compounds. Therefore, we utilised the CD31^+^ cell tubulogenesis assay as a measure reflecting *in vivo* angiogenesis.

We observed that 435S cells promote *in vitro* angiogenesis in a cell number dependent manner. Furthermore, EA>EGCG attenuated this proangiogenic effect. The initial antiangiogenic properties attributed to EA and EGCG (when incubated with non-conditioned media) are distinct from their increasing capacity to suppress the CD31^+^ cell tubulogenesis promoted by increasing numbers of 435S cells, thus suggesting another mode of action. Given that EGCG has been shown to inhibit cancer-associated enzymes such as matrix metalloproteinases (MMPs) (Yamakawa *et al*, 2004) and the proteasome (Kazi *et al*, 2004), we suggest that EGCG and EA inhibition of secreted NDPK-B is another anticancer and antiangiogenic property that can be attributed to these polyphenolic compounds. We propose that EA's potency as an NDPK-B inhibitor and the subsequent reduction of extracellular ATP levels (Yang *et al*, 1994; Malmquist *et al*, 2001) may, in part explain why EA is a more potent inhibitor of CD31^+^ cell tubulogenesis than is EGCG. Known antiangiogenic levels of endostatin did not inhibit NDPK transphosphorylation activity and its diminishing effectiveness in suppressing *in vitro* angiogenesis in response to increasing numbers of 435S cells, further suggests that breast cancer cell-secreted NDPK may play an important role in promoting angiogenesis. One other possible explanation consistent with our hypothesis would be that these compounds also block the activation of nucleotide receptors. However, radioligand binding competition studies with EC P2Y_1_R do not support this possibility (data not shown). Epigallocatechin gallate and EA are potent but non-specific inhibitors of NDPK transphosphorylation activity; therefore, we explored further the extracellular nucleotide aspect of our secreted NDPK angiogenesis model. Supporting this hypothesis, we found that the degradation of ATP and ADP by apyrase suppressed the ability of 435S cell-conditioned media to induce *in vitro* angiogenesis.

We observed decreased tubulogenesis levels with increasing conditioning durations with 435S cells. This negative correlation is not clear mechanistically, but does not argue against the presence of a paracrine and/or autocrine proangiogenic effect *in vivo* since if in the blood stream, such effects would be expected to take place adjacent to endothelium diluted by blood flow and thus would not accumulate but would be replenished by NDPK-B protein secretion.

Nucleoside diphosphate kinase's extracellular role may be underappreciated in carcinogenesis especially in tumour angiogenesis, as indicated by the plethora of studies involving only its intracellular actions. The secretion of NDPK orthologues by intracellular parasites as a possible survival tactic (Gounaris *et al*, 2001; Chopra *et al*, 2003) and the secretion of NDPK by various solid and haematological malignancies (Anzinger *et al*, 2001; Okabe-Kado and Kasukabe, 2003) lead us to propose the pathological role of secreted NDPK in promoting cancer and tumour angiogenesis. Extracellular NDPK in the tumour vasculature would elevate nucleotide signalling and subsequent release of vasoactive factors with angiogenic effects (Ziche and Morbidelli, 2000). In support of our hypothesis, a positive correlation between the regulation of P2Y signalling and various angiogenic properties (Moser *et al*, 2001; Tanaka *et al*, 2004; Kaczmarek *et al*, 2005) is known.

Our results with purified NDPK, (a mixture of NDPK-A and -B), together with the observation that both NDPK isoforms are secreted by various cancers suggests that the transphosphorylation activity of NDPK and not its isoform is crucial. We tested human breast cancer secreted NDPK and observed that NDPK-B depletion from 435S cell-secreted NDPK extract progressively reduced its ability to promote CD31^+^ cell tubulogenesis. The release of NDPK into the incubation buffer is not the result of cell death, since we determined that greater than 10% of cells would need to undergo complete lysis (not apoptosis) during our 90 min collection to obtain similar levels of transphosphorylation activity to NDPK extracts (data not shown). Moreover, when examined after extract collection, cultures are healthy and can be returned to the incubator and observed to be viable. ATP synthase does not account for ATP production as we used an extract devoid of intact cells or membranes. Conditioned media did not contain adenylate kinase activity. Autophosphorylation assays (Backer *et al*, 1993) have been used to monitor NDPK transphosphorylation activity; we employed a phosphoryl transfer assay because it produces kinetic data and can determine relative inhibition. The transphosphorylation activity assay has shown that 435S NDPK-B extract and purified bovine liver NDPK have approximately the same *K*_m_ (∼30 *μ*M) for ADP (Anzinger *et al*, 2001).

We observed that 435S cell-secreted NDPK extract and P2Y_1_R activation induce significant *in vitro* angiogenesis and do so to a similar degree. The use of 10 *μ*M 2MS-ATP or 100 *μ*M ATP in promoting CD31^+^ cell tubule-like formations was maximal, likely due to known desensitisation of P2Y_1/2_ receptors at higher agonist concentrations (Ralevic and Burnstock, 1998). MRS2179 blockade of CD31^+^ cell P2Y_1_R activation stimulated with 435S cell-secreted NDPK extract provides strong evidence that human breast cancer-secreted NDPK reaction product provides a proangiogenic signal via P2YR activation (e.g., ATP). The addition of MRS2179 still allowed minimal angiogenic stimulation over negative control, suggesting that P2Y signalling works in concert with other angiogenic pathway(s) that are yet to be elucidated.

The role of *nm23-H2* in promoting metastasis is supported by the observation that a catalytically inactive mutant of NDPK-B significantly suppressed the lung metastasis of human melanoma cells *in vivo* (Hamby *et al*, 2000). Recent work indicates that activated P2Y_2_R's associate and transactivate vascular endothelial growth factor receptor-2 (VEGFR2) (Seye *et al*, 2004), directly linking nucleotide receptor activation to established tumour angiogenesis signalling (VEGF-VEGFR signalling). P2Y receptor potentiation of VEGFR2 signalling therefore may be essential in describing the angiogenic properties of nucleotides such as ATP. P2Y and VEGF receptors being colocalised to caveolar domains in the cell membrane allows the potential of a substantial proangiogenic signal with small amounts of agonist stimulation (Kaiser *et al*, 2002).

**Figure 6:**
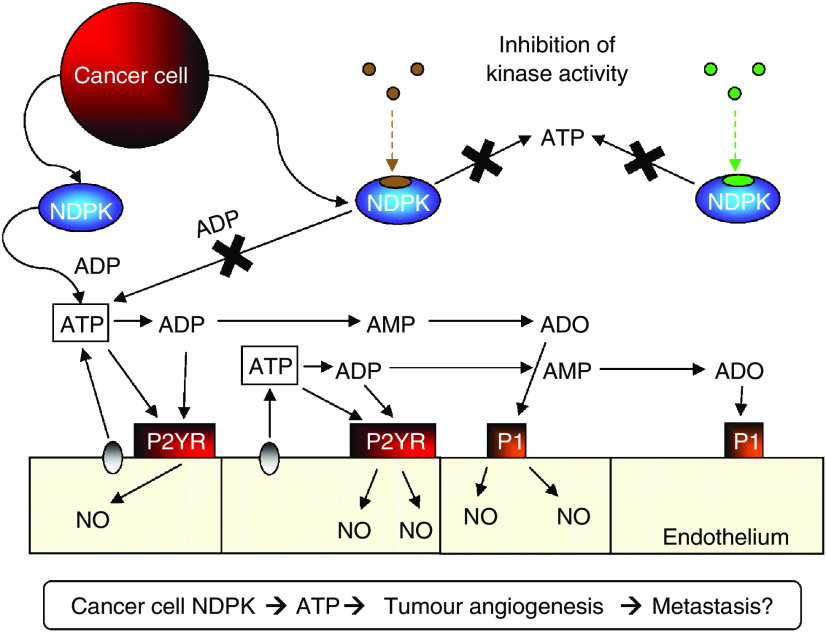
Putative role of extracellular nucleoside diphosphate kinase (NDPK) and P2Y receptor activation in tumour angiogenesis. We hypothesise that breast cancer secreted NDPK-B is an important contributor to promoting angiogenesis and metastasis. Extracellular NDPK would modulate nucleotides such as elevating ATP levels. This scenario would subsequently lead to P2Y purinergic receptor activation above an unknown threshold to produce conditions favorable to pathological angiogenesis.

P2YR signalling is a tumour angiogenesis and metastasis mechanism which 435S cells exploit. Since cells secrete pre-phosphorylated NDPK-B (Anzinger *et al*, 2001), ATP generation locally *in vivo* may require little substrate and donor (a triphospho-nucleotide such as GTP) may be intracellular. Our model predicts two spatially distinct yet conceptually dependent mechanisms: NDPK recycling of ADP back to ATP in both arteries and capillaries. Nucleoside diphosphate kinase in the arteries may elevate local nucleotide ATP concentrations to produce P2YR-mediated vasodilation and anti-platelet aggregation, advantageous to the transit of cancer cells to secondary sites. Nucleoside diphosphate kinase secretion in capillaries may elevate local nucleotide concentrations and produce nucleotide receptor (e.g., P2Y) mediated angiogenesis (Figure 6). As an example supporting this hypothesis we provide evidence that extracellular NDPK induces EC tubulogenesis predominately via P2Y_1_R. Elevated levels of ATP and subsequent P2YR activation will release additional ATP to prolong the angiogenic effect (Yang *et al*, 1994). The constant elaboration of NDPK-B by 435S cells facilitates the chronic and increased activation of P2Y signalling, producing pathological angiogenesis in tumour vasculature. Since the NDPK-B enzyme can be expressed as both an ecto- or exo-enzyme and help to regenerate 8–12% of ADP in blood back to ATP (Buxton *et al*, 2001), it may play a significant role in angiogenic regulation especially in the setting of apoptosis and necrosis associated with tumour growth as a source of triphospho-nucleotide donor.

We are aware that 435S carcinoma cells exhibit both breast cancer and melanoma characteristics (Sellappan *et al*, 2004). This fact strengthens our observation that NDPK-P2Y angiogenic signalling may be utilised in a broad range of tumours. P2Y receptor signalling has also been observed to modulate proliferation of various carcinomas in a positive, and negative fashion (White and Burnstock, 2006). One possible explanation is that these differing effects on proliferation reflect which cancers pathologically secrete NDPK.
